# Let there be laser light

**DOI:** 10.1038/s41467-018-04302-9

**Published:** 2018-05-16

## Abstract

Today, on the 16th May, we celebrate the first International Day of Light, which UNESCO proclaimed late last year. We celebrate the central role light and light-based technologies play in our lives and consider how they can be beneficial to humanity through sustainable lighting, renewable energy and improved health care.

Sixteenth of May—the International Day of Light—marks the day Theodore Maiman saw the first working laser in his laboratory in 1960^1^; as his wife Shirley related in an interview with the New York Times, he came home and simply told her: 'My experiment worked today'. At the time—4 years after Maiman’s experiment—there were few applications for the new technology and he described it in the same interview as 'a solution seeking a problem'—a phrase that was uttered by many during the 1960s, according to Charles Townes. And it took indeed over 20 years for laser technologies to become a market.

Unsurprisingly, science fiction writers adopted lasers much more quickly for their stories to replace the ever-popular heat guns and death rays. Even today, when the laser technology market is worth more than US$ 10 billion (and still growing), many people first encounter lasers in comic books or science fiction and action movies. And a favourite trick of movie villains seems to be threatening the hero with a laser beam that easily cuts through the table the hero is strapped to while it comes closer and closer. Although unlikely, it is not an entirely impossible use of a laser; indeed, cutting and welding are the main applications of lasers in factories.

**Figure:**
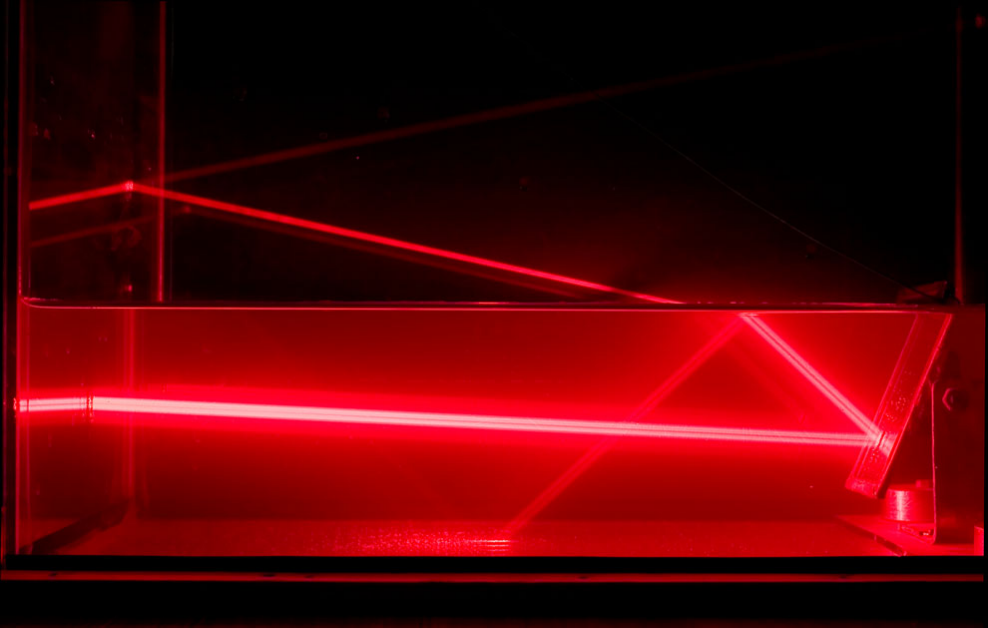
dra_schwartz/E+/Getty

However, far from being only a powerful light source that can cut or burn, the laser is an excellent example of how fundamental research can lead to a plethora of applications and of the abundance of optical technologies in the twenty-first century world. It perfectly illustrates the interdisciplinary nature of the International Day of Light because it is a key enabling technology in research, industry and even art.

Although most people will never see an industrial laser in action, we all use laser technology every day: lasers power the internet (or at least the optical communication behind fibre broadband); lasers measure distances on building sites and car speeds on motorways; lasers read the barcodes at the check-out in supermarkets; lasers read CDs, DVDs and BluRay discs; laser printers can be found in offices and laser projectors in modern cinemas.

Even modern medicine uses lasers as an integral part of some diagnostics, surgery and treatment. Here, laser eye surgery is probably the best known, but these days, laser scalpels can deliver precise cuts to a diverse range of tissues, including in neurosurgery. Laser light can be directed through the fibre bundles inside an endoscope to a specific site in the body to perform surgery or to destroy tumour cells by heating, often assisted by a previously injected photosensitizer. Before such an operation, a laser might even be used to diagnose the cancer by imaging a tissue sample taken in a minimally invasive biopsy.

Back in the lab, imaging is also one of the many techniques where lasers are used in research, both in the physical and in the life sciences: fluorescence microscopy, particularly in the guise of super-resolution microscopy, has dramatically improved our understanding of sub-cellular biological structures and processes. Laser spectroscopy has become a powerful tool for material characterisation, time-resolved laser spectroscopy has revealed the dynamics of many physical systems, developing our understanding of materials and fundamental physics. To study such properties at the microscale, one can use laser lithography to fabricate three-dimensional (3D) microscopic structures in a way similar to the popular 3D printers. Lasers can even help us understand the brain as they can be used to control and manipulate the activity of neurons in a targeted manner, enabling the field of optogenetics.

Despite the widespread use and the maturity of laser-based technology, the book on laser physics is far from written and even further from being shelved. Instead of merely optimising the device performance of lasers employed in market technologies, researchers continue to investigate the intricate details of the lasing process and develop completely novel laser principles that go beyond the engineering of gain materials and cavity designs.

Random lasers, for example, abolish the conventional closed cavity altogether and the optical feedback that amplifies the stimulated emission is instead provided by multiple scattering in a disordered medium. For this reason, the emission from random lasers is more complex than the well-controlled modes of conventional lasers; random lasers can emit at many different wavelengths and in many different directions rather than emitting the well-defined beam we have come to associate with lasers. While this presents challenges for applications, their randomness makes the complex dynamics of this laser class an interesting fundamental question.

Although laser oscillations which signify the build-up of lasing, and other dynamics on picosecond timescales have been known for some time, the real-time measurement of ultrafast dynamics, for example, of the mode competition in the build-up of mode-locking, has only recently been possible. These real-time methods also allow the investigation of complex and chaotic dynamics in laser fluctuations. To date, such studies have been largely confined to solid-state and fibre lasers, but expanding such investigations to other laser systems promises further insight into the properties of the diverse laser family.

In the quest for miniaturisation, a macroscopic mirror cavity can be replaced with a dielectric microresonator. Microlasers studies have recently been invigorated by the development of non-Hermitian photonics, where loss and gain in a structure are used to achieve or break parity-time (PT) symmetry. This approach leads to stable single-mode emission. Since it is often realised using balanced losses instead of loss and gain, it can lead to the counter-intuitive onset of lasing with an overall increase of losses. PT-symmetric lasing is not confined to microstructures and has also been shown with an optical fibre network, but only PT-symmetric microlasers have very recently been combined with topological effects to form hybrid topological lasers, where the lasing mode is also topologically protected, that is, immune to small perturbations like scattering.

Further miniaturisation takes us to the nanoscale, where a theoretical proposal in 2003 triggered research into spasers—and more generally into plasmonic lasers—and other nanolasers. Over a decade later, both plasmonic and dielectric nanolasers have been realised, although laser dynamics of nanolasers are not widely studied nor has mode-locking been achieved yet. Nonetheless, nanolasers have already shown promise in applications, for example, to improve photothermal cancer therapy.

However, the biocompatibility of metal nanoparticles remains a largely open question. Biolasers (and random biolasers) made entirely from biological or biocompatible materials could fill this niche; a biolaser might even be a living cell. Most laser dyes are, however, toxic and need to be replaced with bioderived or at least biocompatible fluorophores like green fluorescent protein. Biolasers have so far not been developed beyond a proof of principle and further work is needed to make them fully biocompatible and controllable before such devices can be tested in their intended environment—tissues and living organisms.

Fifty-eight years after Maiman’s ruby laser, lasers have evolved into a diverse family of devices striving to occupy every possible technological niche. They have become an integral part of modern technology and it is hard to imagine our daily life without them. At the same time, new methods and new classes of lasers continue to open new avenues of investigation and provide a compelling subject of research. *Nature Communications* is looking forward to seeing many such exciting developments realised in the years to come.
